# Exploration of wearable sensor measures associated with panic attacks differs across mental health conditions

**DOI:** 10.3389/fdgth.2026.1764371

**Published:** 2026-04-20

**Authors:** Dheeraj Dhanvee Kairamkonda, Ellen W. McGinnis, Matthew Price, Johanna E. Hidalgo, Julia Kim, Jordan Llorin, Kathryn Stanton, Laura S. P. Bloomfield, Mikaela Fudolig, Jennifer Ha, Natalie Noble, Josh Cherian, Guido Mascia, Nathaniel S. O'Connell, Jason Fanning, Peter Sheridan Dodds, Christopher M. Danforth, Ryan S. McGinnis

**Affiliations:** 1 Center for Remote Health Monitoring, Wake Forest University School of Medicine, Winston-Salem, NC, United States; 2 Department of Biomedical Engineering, Wake Forest University School of Medicine, Winston-Salem, NC, United States; 3 Department of Social Sciences and Health Policy, Wake Forest University School of Medicine, Winston-Salem, NC, United States; 4 Department of Pediatrics, Wake Forest University School of Medicine, Winston-Salem, NC, United States; 5 Project LEMURS (Lived Experience Measured Using Rings), University of Vermont, Burlington, VT, United States; 6 Department of Psychological Science, University of Vermont, Burlington, VT, United States; 7 Vermont Complex Systems Institute, MassMutual Center of Excellence for Complex Systems and Data Science, University of Vermont, Burlington, VT, United States; 8 Department of Computer Science, University of Vermont, Burlington, VT, United States; 9 Department of Mathematics and Statistics, University of Vermont, Burlington, VT, United States; 10 Department of Biostatistics and Data Science, Wake Forest University School of Medicine, Winston-Salem, NC, United States; 11 Department of Health and Exercise Science, Wake Forest University, Winston-Salem, NC, United States; 12 Santa Fe Institute, Santa Fe, NM, United States

**Keywords:** adverse childhood experiences, generalized linear mixed-effects models, mental health diagnosis, odds ratios, Oura Ring, panic attacks

## Abstract

Panic attacks (PAs) are acute anxiety episodes that are pervasive, with one in 10 individuals having experienced a PA in the past year. PAs impair daily functioning and are associated with an increase in emergency room visits and suicide attempts. Despite their impact, the unpredictable nature of PAs makes them challenging to manage. PAs are transdiagnostic, occurring in individuals across and without a mental health diagnosis. However, prior work has largely focused on PA indications within individuals with panic disorder. This study identifies PA risk factors from over 6 months of passive sensing data recorded by Oura Rings in 182 young adults with and without adverse childhood experiences and psychiatric diagnoses, beyond just panic disorder. Our findings reveal that changes in Oura Ring–derived measures are associated with next-day PAs, with distinct associations observed across different mental health diagnoses. For individuals with panic disorder, the likelihood of PA increases with time spent inactive. For those with depression, the likelihood of PA increases with decreased variation in nightly respiratory rate, decreased rapid eye movement sleep, and increased time spent in high-intensity activity. For those without a mental health diagnosis, the likelihood of PA increases with decreased heart rate variability. Data aggregation window sizes that capture the associations with PA risk vary by diagnosis and the type of feature, suggesting that cumulative physiological patterns from windows up to 7 days before a PA contribute to onset. These findings point to the possibility that continuous monitoring of panic attack risk could one day support preventive mental health intervention.

## 1 Introduction

Panic attacks (PAs) are sudden and seemingly unexpected episodes of intense anxiety, accompanied by uncomfortable somatic symptoms such as hyperventilation, tachycardia, chest pain, nausea, sweating, and feeling out of control of one's own body (1). Over 28% of US adults have experienced at least one PA in their lifetime, and one in 10 has experienced a PA in the past year (2). Panic attacks are transdiagnostic, occurring in individuals across mental health conditions and even those without any diagnoses (2). PAs may act as a harbinger of autonomic, emotional (3), and cognitive (4) dysregulation commonly layered over adverse childhood experiences (5) and depression and associated with suicidal attempts (4). Therefore, not surprisingly, PAs are associated with poorer wellbeing (6), missed days of work (7), substance misuse (8), increased Emergency Department visits (9, 10), and suicide (11).

The unpredictable nature of panic attacks (PAs) makes them particularly challenging to manage. Evidence-based treatments have traditionally consisted of pharmacological approaches and clinician-delivered psychotherapies [see (12–15)]; however, these interventions are often associated with significant barriers, including cost and limited access to trained providers. In response, there has been growing interest in lower-barrier, brief preventive strategies that individuals can plausibly self-administer (16, 17). Individuals who experience PAs have expressed interest in tools that support prevention and early coping outside of clinical settings (18–20). As with other episodic conditions, the effectiveness of such self-managed approaches is highly dependent on timing, and support is most beneficial when delivered during periods of heightened vulnerability, when individuals are most receptive (21). Despite this, relatively little is known about the short-term behavioral and physiological contexts that precede PAs, constraining the development of timely and adaptive preventive interventions.

Wearable sensing offers a scalable approach for identifying short-term changes in autonomic and behavioral function that may precede panic attacks. Across wearable-based panic attack forecasting studies, commonly informative inputs include heart rate and heart rate variability, respiratory features, sleep parameters, and activity levels, signals that reflect sympathetic–parasympathetic balance, hyperarousal and respiratory dysregulation, and disruptions in recovery and daily routines (22–27). Much of the foundational evidence for these markers, however, has been derived from laboratory-based studies or from protocols relying on intensive, burdensome instrumentation (e.g., multiple wired sensors) (24, 28), limiting ecological validity and scalability. Consequently, there remains a need to examine whether similar signals can be detected using low-burden, consumer-grade wearables in naturalistic settings.

Although objective markers specific to PA vulnerability are still understudied, insight can be drawn from related research on emotional and physical stress processes. Self-reported triggers (19) among individuals experiencing PAs most commonly include emotional overwhelm (36%), interpersonal conflict (15%), and physical discomfort (e.g., headache; 14%). Such work documents characteristic changes in both behavior (e.g., reduced physical activity, increased insomnia) (25–27) and physiology (e.g., decreased heart rate variability, increased respiratory rate) (29–31), which may provide a useful framework for characterizing the contexts that precede PAs. Examining these domains using passively collected wearable data may therefore help identify candidate markers of near-term PA risk.

At the same time, heterogeneity in mental health history complicates PA prediction (19). Underlying mental health conditions and related experiences such as trauma exposure are known to influence baseline sleep, activity, and physiological functioning (32–38), as well as reactivity to stressors (32–38). For example, depressive disorders have been associated with blunted or prolonged physiological stress responses (39), potentially driven by rumination (40), whereas anxiety-related conditions are often characterized by heightened autonomic reactivity across multiple indices [e.g., electromyography, cortisol see (41, 42)]. Behavioral responses to stress may also diverge by diagnosis; for instance, individuals with depression may increase sleep duration under stress, whereas individuals without a mental health diagnosis may exhibit insomnia (43, 44). As a result, the direction, magnitude, and timing of wearable-derived features associated with PA risk may vary across individuals as a function of mental health history. Understanding this heterogeneity is critical for developing interpretable PA forecasting models and for informing adaptive, personalized prevention strategies. Notably, the majority of existing PA research using wearable or physiological data has focused exclusively on individuals diagnosed with panic disorder (45–47), despite evidence that this group represents only a subset of those who experience PAs.

In this study, we examine the feasibility of using Oura Ring–derived behavioral and physiological data to identify markers associated with next-day PA risk, with the goal of generating PA-specific hypotheses for future research. We leverage longitudinal data from a sample of college students, a population with a high prevalence of mental health concerns, who reported experiencing at least one PA during an academic-year wellness study. Given prior evidence that stress responsiveness and autonomic regulation are moderated by mental health history, and to improve interpretability in this exploratory context, we stratify analyses in two ways: by the presence vs. absence of adverse childhood experiences (ACEs), and by diagnostic status (no diagnosis, depression, panic disorder). Using wearable-derived metrics previously linked to stress and panic vulnerability, we examine sleep [e.g., sleep stage distribution, duration, and timing (48)], physiological signals [e.g., respiratory rate and variability, heart rate, and heart rate variability (18, 24, 28, 33, 49)], and activity patterns [e.g., time spent across activity levels and step count (50, 51)] in relation to PA risk and assess whether these associations differ across mental health groups (52). Although exploratory due to relatively small subgroup sizes, this work is among the few studies to examine wearable-derived PA risk markers in individuals both with and without panic disorder. Identifying short-term, passively measured indicators of elevated PA risk may ultimately support the delivery of brief, self-managed preventive interventions during periods of heightened vulnerability, while minimizing user burden and alert fatigue.

## 2 Methods

### 2.1 Procedure

This work is part of the Lived Experiences Measured Using Rings Study (LEMURS), wherein the aims included understanding objective metrics underlying wellness in college students. This study was approved by the University of Vermont Institutional Review Board, and all participants signed informed consent forms. In the LEMURS, first- and second-year undergraduate students were recruited to participate in a study about wellness through flyers and tabling events across campus. Eligibility criteria were (1) being between the ages of 18–24, (2) being a full-time first- or second-year student at the University of Vermont, and (3) owning an iPhone 6S/SE or newer to use the Oura App. All participants were provided with an Oura Ring, which is a wearable biometric device that records heart rate, heart rate variability, body temperature, respiration rate, sleep latency, sleep efficiency, sleep duration, and sleep staging among other metrics. The validity of Oura's sleep and activity measurements have been accepted throughout general literature, especially in comparison with other available commercial devices (53–57). The participants were instructed to wear the ring at all times, including during sleep, and their Oura Ring information was accessible to them via the Oura app. The participants completed a baseline assessment that included a demographic questionnaire. They were then randomly divided into three groups and assigned to rotating 21-day intervals during which they received daily surveys regarding panic and stressors and then 42 days where they did not receive any survey. We designed the study with rotating daily survey sprints to reduce participant burden and minimize reporting fatigue, while also enhancing the likelihood of receiving responses from the participants across the study period (58, 59). Once one group's 21-day interval of daily surveys was over, a new group started their 21-day interval of daily surveys. This was repeated four times throughout the academic year, for a total of 252 days. Each participant received four sets of 21-day interval daily surveys, for a total of 84 daily surveys per participant throughout the study period.

**Figure 1:**
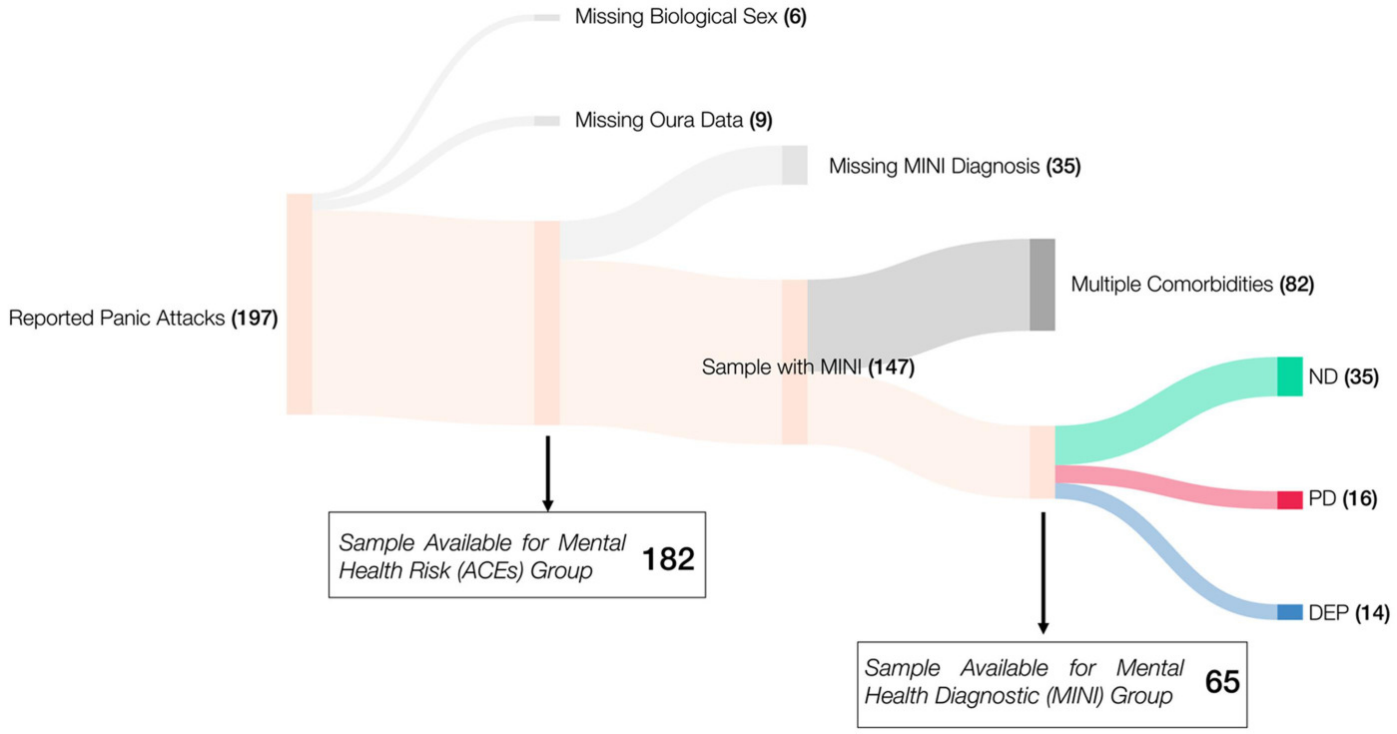
Participant selection and distribution into mental health risk (ACE) and mental health diagnostic (MINI) groups. The initial sample included 197 participants who reported at least one panic attack. Participants missing sex information (*n* = 6) or Oura data (*n* = 9) were excluded, leaving 182 (ACE: 154; None: 28) participants for the Mental Health Risk (ACEs) group analysis. From this group, those missing MINI diagnoses (*n* = 35) were excluded, resulting in 147 participants, of whom 82 had multiple comorbidities, resulting in 65 participants available for the Mental Health Diagnostic (MINI) group analysis. Of this sample, there were 35 participants with no mental health diagnosis (ND), 16 with panic disorder (PD, alone and with comorbid anxiety and/or ADHD), and 14 with depression (DEP). All participants with a diagnosis (PD or DEP) had ACEs, and in those with no diagnosis, 24 participants had ACEs, while 11 participants did not. In the Sankey plot, flows representing exclusion criteria are shown in light gray, and dark gray represents the samples that were not considered in the analysis because of the presence of multiple comorbidities.

Of the 525 participants enrolled in the LEMURS during the 2023–2024 academic year, 197 (38%) endorsed experiencing at least one PA. Fifteen participants were excluded for missing data (missing biological sex, *n* = 6; Oura data, *n* = 9), leaving data from 182 participants for analysis. The participants were first grouped by a known risk factor for developing mental health disorders, adverse childhood experiences [ACEs (5)]: (presence, *n* = 154; absence, *n* = 28; total of 507 PA days from 28,389 participant-days; almost 84% (*n* = 127 out of 152) of females reported to have ACEs, while 90% (*n* = 27 out of 30) of males reported to have ACEs; more details in Supplementary Table S1 and Supplementary Figure S3). Next, the participants were grouped by the presence of specific mental health diagnoses, which exhibit differential rates of PAs (60). Mental health diagnosis was determined via administered diagnostic interviews, from which 35 participants were missing. We focused on three groups for analyses, given their known differential risk of PAs: those with depression only (“DEP,” *n* = 14), those with panic disorder alone and with comorbid anxiety and/or ADHD (“PD,” *n* = 16), and those with no diagnosis (“ND,” *n* = 35), with a total of 148 PAs from 10,643 participant-days, where the panic attack rate for ND was 4.8%, meaning 1 PA every 21 days, while for PD, it was 5.6%, and for DEP, it was 5.8%, meaning approximately 1 PA every 18 days for both the groups. Participants with generalized anxiety disorder only, ADHD only, and/or comorbidities with depression were excluded from analyses (*n* = 85, see details in Supplementary Figure S4) because of limited sample size per group (*n* < 10) or for exhibiting comorbidities. Although research on the association of PAs with diagnostic comorbidities is critical for generalizability, given the insufficient literature on association between comorbidities and single diagnoses on physiology generally (42, 61), we chose to exclude them for this exploratory analysis to better develop hypotheses for future work on comorbidity. Sixty-five participants were available for the mental health diagnostic group analysis. All participants who had DEP or PD reported ACEs (Figure 1). Demographic characteristics of the sample, separated by analysis groups, are reported in Table 1. Distribution of participants by racial/ethnic group is presented in Supplementary Table S2.

**Table 1:** Demographics of the sample, Oura Ring wear times, and panic attack days within each group.

Group	*N*	Sex (female)	Age (mean)	Race and ethnicity (white, non-Hispanic)	Oura wear days	Panic attack days
Mental health risk groups						
None	28	25 (89%)	18	23 (82%)	163	40
ACES	154	127 (82%)	18	137 (89%)	159	463
Mental health diagnostic groups						
No diagnosis (ND)	35	27 (77%)	18	30 (86%)	166	67
Depression (DEP)	14	10 (71%)	18	11 (79%)	161	36
Panic disorder (PD)	16	13 (81%)	18	15 (94%)	163	45

### 2.2 Measures

#### 2.2.1 ACE survey

We used the criteria described in Supplementary Table S1 to group someone as having an ACE (62) from a baseline self-report survey. Internal consistency of the 10 ACE items used was acceptable (Cronbach's *α* = 0.69).

#### 2.2.2 Mental health diagnostic interviews

The Mini-International Neuropsychiatric Interview (M.I.N.I.) (63) is a short structured diagnostic interview administered by a research team to participants at an in-person visit during the academic year. Diagnostic scoring criteria were followed to obtain diagnoses for each participant.

#### 2.2.3 Daily surveys

Items included a checklist of potential stressors, and whether they experienced a panic attack, and an open response option to include what their biggest stressor was each day. For these analyses, at all days wherein a panic attack occurred, responses to the biggest concern were coded, resulting in 171 coded responses: 94% (33 of 35) of ND individuals (79 days of responses), 86% (12 of 14) of DEP (37 days of responses), and 100% (16 of 16) of PD (55 total responses).

#### 2.2.4 Oura Ring data

Daily summaries of sleep and activity data from the Oura Ring were used in the analysis. Note that the variables/data retrieved from the Oura Ring include two kinds of measurements: “raw measures” [e.g., heart rate, heart rate variability (HRV), and steps] that are highly interpretable in nature and “scores” (e.g., sleep score, sleep latency score, and sleep alignment score) that are composite metrics generated by Oura's proprietary algorithms. Each observation represents an average summary statistic of all the sleep metrics from the night and the average summary of all the activity metrics from the day. Missing data were filled using linear interpolation, provided there were three or less days between observations. Statistical analyses were repeated considering filling gaps up to 2 days and 1 day. Selected features remained significant, and the directions of association were consistent across levels of interpolation (Supplementary Table S3). All metrics available from the Oura Ring are listed in Table 2.

**Table 2:** OURA raw measures and scores.

Sleep/activity	OURA raw measures/Scores	Features	Description	Units
Activity	OURA raw measures	Active calories burned	Energy consumed during the day while doing physical activity	Kilocalories
		Total calories burned	Active calories burned plus the basal metabolic rate	Kilocalories
		Daily movement	Daily activity converted to equivalent walking distance	Meters
		^a^ **Steps**	Total number of steps registered during the day	Steps
		Non-wear time	Number of minutes during the day that the user did not wear the ring	Minutes
		^a^ **Time resting**	Number of minutes during the day spent resting (e.g., lying down and sleeping)	Minutes
		^a^ **Time inactive**	Number of minutes during the day spent inactive (e.g., sitting and standing)	Minutes
		^a^ **Time in low intensity**	Number of minutes during the day with low-intensity activity (e.g., housework)	Minutes
		^a^Time in medium intensity	Number of minutes during the day with medium-intensity activity (e.g., walking)	Minutes
		^a^ **Time in high intensity**	Number of minutes during the day with high-intensity activity (e.g., running)	Minutes
		^a^METs	Average MET level during the day	MET
		METs during inactivity	MET minutes recorded during the inactive minutes of the day	MET minutes
		METs during low-intensity activity	MET minutes recorded during the low-intensity activity minutes of the day	MET minutes
		METs during medium-intensity activity	MET minutes recorded during the medium-intensity activity minutes of the day	MET minutes
		METs during high-intensity activity	MET minutes recorded during the high-intensity activity minutes of the day	MET minutes
	OURA scores	^a^Activity score	Estimate of how well recent physical activity has matched user needs	Range (1–100)
		^a^Stay active score	Estimate of how well the user has avoided inactivity during the day	Range (1–100)
		^a^Move every hour score	Estimate of how well the user has avoided long periods of inactivity during the day	Range (1–100)
		Score meet daily targets	Estimate of how often the user has reached daily activity targets in the last 7 days	Range (1–100)
		Score training frequency	Estimate of how often the user has exercised in the last 7 days	Range (1–100)
		Score training volume	Estimate of how much the user has exercised in the last 7 days	Range (1–100)
		Score recovery time	Estimate of how much recovery time the user has obtained in the last 7 days	Range (1–100)
		^a^Number of inactivity alerts	Number of continuous inactive periods of 60 min or more during the day	Range (1–100)
Sleep	OURA raw measures	Sleep-onset latency	Time between the start of the sleep period and the first 5 min of persistent sleep	Seconds
		^a^ **Bedtime offset**	Time when the sleep period starts	Seconds
		^a^ **Wake-up offset**	Time when the sleep period ends	Seconds
		Time midpoint	Time corresponding to the midpoint of the sleep period	Seconds
		^a^Time awake	Total amount of awake time during the sleep period	Seconds
		^a^Time in light sleep	Total amount of light (N1 or N2) sleep during the sleep period	Seconds
		^a^ **Time in REM sleep**	Total amount of REM sleep during the sleep period	Seconds
		^a^Time in deep sleep	Total amount of deep (N3) sleep during the sleep period	Seconds
		^a^Total sleep time	Total amount of sleep during the sleep period (REM + Light + Deep)	Seconds
		Total sleep duration	Total duration of the sleep period (REM + Light + Deep + Awake)	Seconds
		Sleep efficiency	Percentage of the sleep period spent asleep	Range (1–100)%
		^a^Restlessness	Percentage of the sleep period spent moving	%
		Got up frequency	Number of times the user got up during the sleep period	Unit: increments of 1
		Wake-up frequency	Number of times the user woke up during the sleep period	Unit: increments of 1
		^a^Temperature deviation	Skin temperature deviation from the long-term temperature average during sleep	Celsius
		Temperature trend deviation	Skin temperature deviation from the weighted 3-day rolling temperature average	Celsius
		^a^ **Breath rate**	Average breathing rate during the sleep period	Breaths per minute
		^a^ **Breath variation**	Variation in breathing rate during the sleep period	Breaths per minute
		^a^ **HRV (RMSSD)**	Average heart rate variability during the sleep period calculated using the RMSSD method	Milliseconds
		^a^Average heart rate	Average heart rate during the sleep period	Beats per minute
		Lowest heart rate	Lowest HR during the sleep period	Beats per minute
		Lowest HR time offset	Time when the lowest HR was recorded	Seconds
	OURA scores	^a^Sleep score	Estimate of overall sleep quality during the sleep period	Range (1–100)
		^a^Sleep latency score	Estimate of the contribution of sleep-onset latency to sleep quality	Range (1–100)
		^a^Sleep alignment score	Estimate of the contribution of circadian alignment to sleep quality	Range (1–100)
		^a^REM sleep score	Estimate of the contribution of REM sleep time to sleep quality	Range (1–100)
		^a^Total sleep score	Estimate of the contribution of total sleep time to sleep quality	Range (1–100)
		^a^Deep sleep score	Estimate of the contribution of deep (N3) sleep time to sleep quality	Range (1–100)
		^a^Sleep efficiency score	Estimate of the contribution of sleep efficiency to sleep quality	Range (1–100)

Metrics used in the analysis are indicated with an (^a^), and those used in the multivariable analysis are in bold. Summary statistics were applied to these metrics for the reported analyses.

To identify the trends preceding a PA, we applied sliding windows of lengths 3–7 days with a step size of 1 day. From each window, we extracted statistical features such as mean, standard deviation, and 5th, 50th, and 95th percentiles for each Oura metric. Windows shorter than 3 days were not considered because they do not provide stable estimates of variance. The resulting feature vectors were labeled as preceding a PA if the day directly following the window corresponded to a PA event. All features were standardized using participant-specific means and standard deviations. This process produced a large number of features, and we made efforts to reduce the number of features considered by removing those that did not have an expected relationship to PAs or were highly collinear (correlation >0.80, Table 2).

### 2.3 Statistical analysis

Generalized linear mixed models (64) (GLMMs) were used to identify associations between Oura Ring metrics and next-day participant-reported PAs. GLMMs are well-suited for handling longitudinal data with repeated measures and can account for within-subject correlations by including random effects, which are essential for our data considering the multiple observations per participant across different time points. Biological sex was associated with PA frequency and thus was included as a covariate in subsequent analyses.

After aggregating data from the Oura Rings, we performed a three-step analysis approach. First, univariate GLMMs were applied for each mental health risk and diagnostic group where we fit a model to examine the relationship between each feature derived from the Oura Ring data and PAs controlling for biological sex and using participant ID as a random effect (random intercept). The significance of the relationship was assessed by using *p*-values (*p* < 0.05), estimated coefficients, and ORs with 95% confidence intervals (CIs). Given the exploratory nature of our analysis, we did not apply false discovery rate (FDR) correction to the features evaluated in the univariate models. Instead, we adopted a conservative two-step procedure: (1) we first identified features that were significant in the univariate analyses and then further down-selected them using LASSO regression, with the penalty parameter chosen using the 1-SE rule to guard against Type I error; and (2) the retained features were entered into the multivariable GLMMs for each diagnostic group, where significant associations were again identified using *p*-values adjusted for multiple comparisons, when appropriate, via the Benjamini–Hochberg method with an FDR threshold of 0.05.

To validate the assumptions of the GLMMs, we conducted diagnostic tests on the residuals for each multivariable model applied across the panic disorder, depression, and no diagnosis groups where we tested for normality [Kolmogorov–Smirnov (KS)], overdispersion, and outliers in the residuals and found the assumptions to hold true.

## 3 Results

### 3.1 Risk factors of panic attacks differ by ACEs

**Figure 2:**
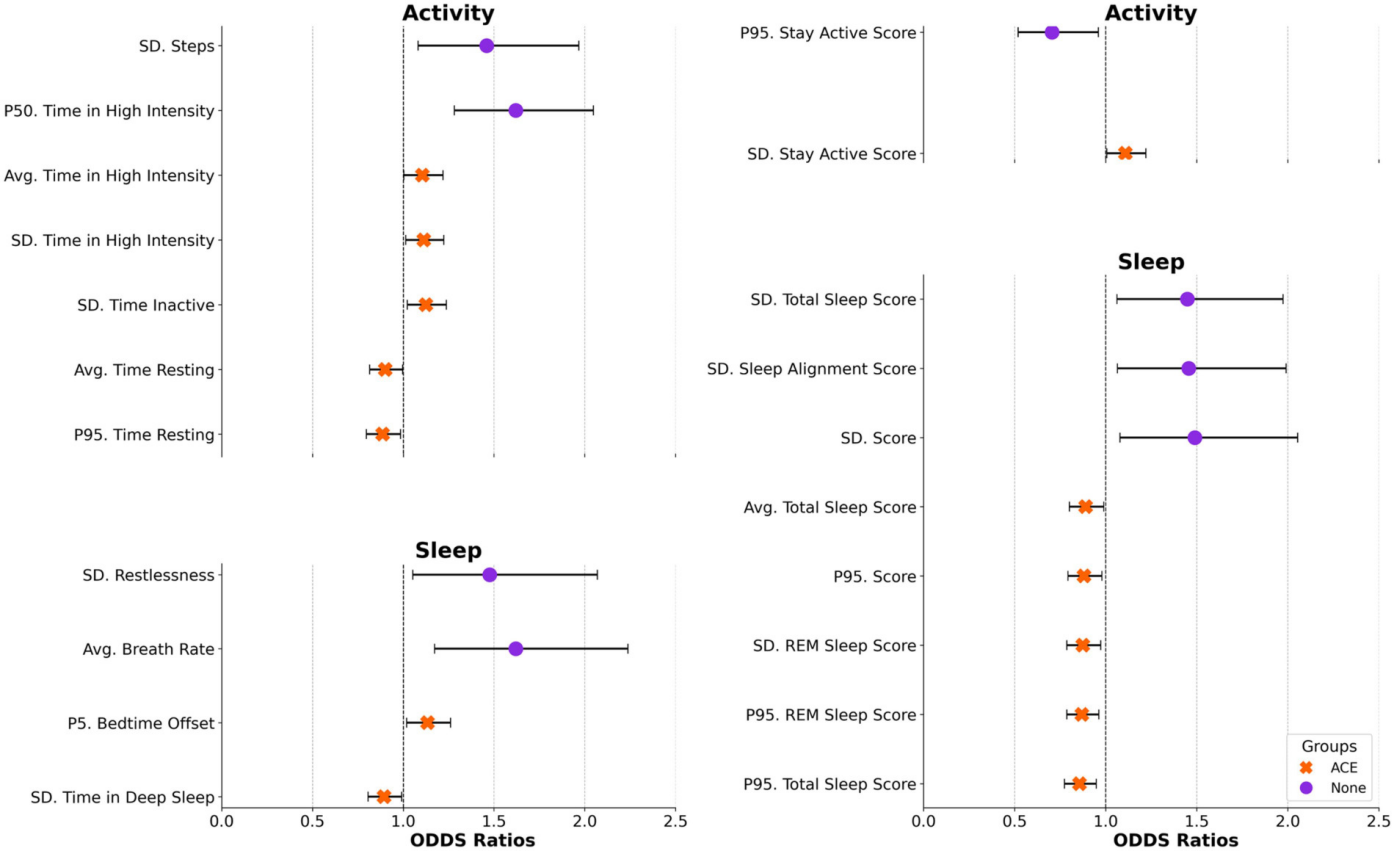
Associations between features and panic attack risk across mental health risk groups. The highest odds ratios (OR) for each significant (*p* < 0.05) feature across the aggregation windows (3–7 days) prior to a panic attack are reported (top) for each mental health risk group (ACE—orange, None—purple) (Avg, mean/average; SD, standard deviation; P5, 5th percentile; P50: 50th percentile; P95: 95th percentile).

Almost twice as many features (*n* = 13 vs 8) were significantly associated with PAs for those with ACEs vs. those without (Figure 2). Of the 11 raw measures that were significantly associated with PA onset, most were unique to each group. However, heightened PA risk was related to a construct of less time spent in high-intensity physical activity for both groups (average vs median time for those with vs without ACEs). For those without ACEs, average breath rate had the highest OR such that a one standard deviation (SD) increase in breath per minute during sleep was related to a 62% increase in risk for a PA. For those with ACEs, ORs were modest, and no feature surpassed a 20% change in PA risk. Ten Oura scores were significantly associated with PAs, each unique by group. For those without ACEs, a standard deviation of the Sleep Score had the highest OR such that a one SD increase in the variation of the score was related to a 49% increase in risk for a PA. For those with ACEs, ORs were again modest, and no feature surpassed a 20% change in PA risk. Supplementary Figure S1 provides additional information about each feature's significant association with PA across different data aggregation windows across mental health risk groups.

### 3.2 Risk factors of panic attacks differ by mental health diagnosis

**Figure 3:**
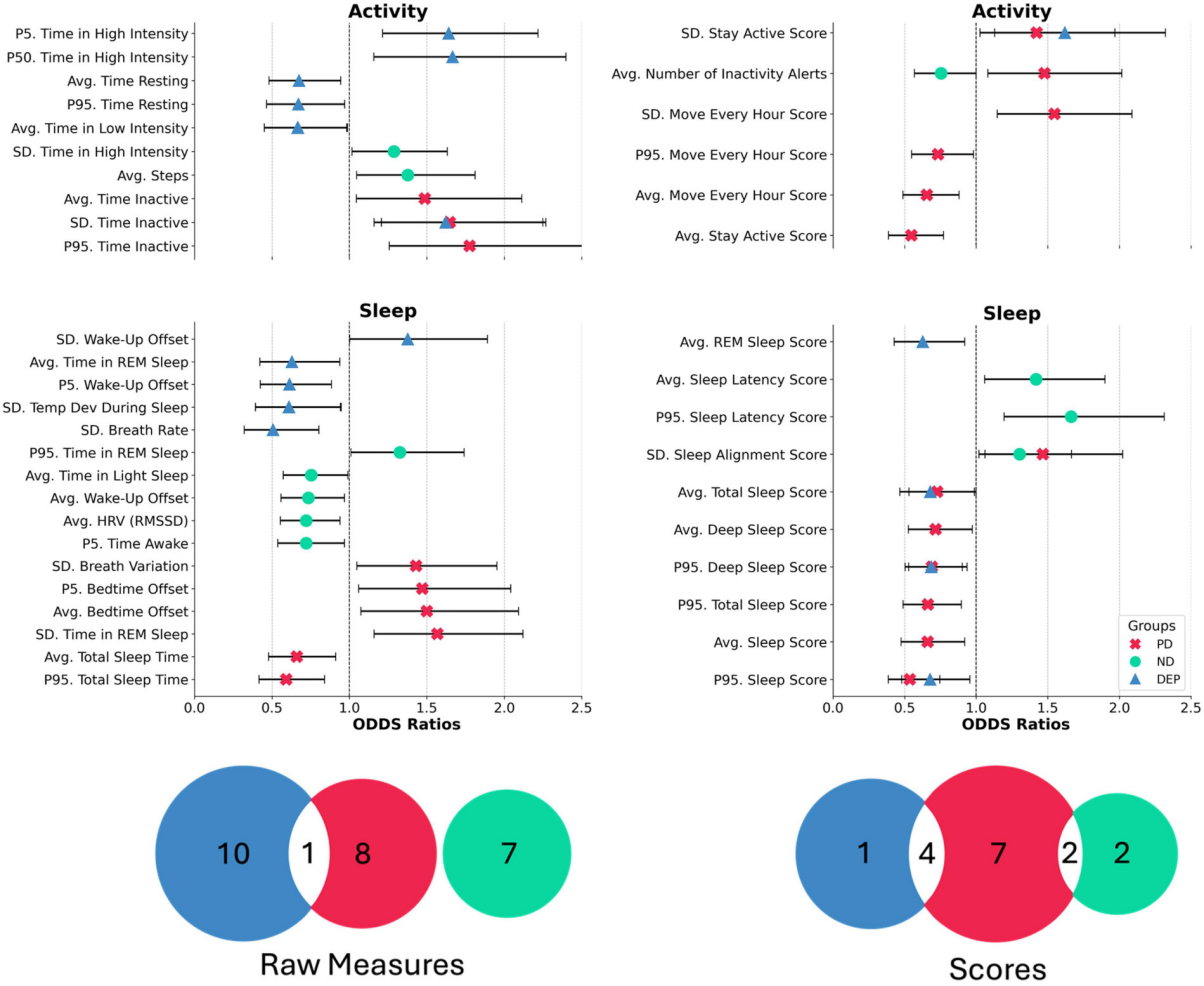
Associations between wearable features and panic attack risk across mental health diagnosis. The highest odds ratios (OR) for each significant (*p* < 0.05) feature across the aggregation windows (3–7 days) prior to a panic attack are reported (top) for each mental health diagnosis group (ND—green, DEP—blue, PD—red). The Venn diagrams (bottom) indicate limited overlap of associated features between mental health diagnosis groups in both raw measures and scores from Oura (Avg, mean/average; SD, standard deviation; P5, 5th percentile; P50, 50th percentile; P95, 95th percentile).

As shown in Figure 3, more features were associated with PAs for those with PD (*n* = 21, ORs: 0.54 – 1.80) than DEP (*n* = 16, ORs: 0.51 – 1.67) or ND (*n* = 11, ORs: 0.72 – 1.66). Most (84%) significant features were unique to a diagnosis. Within the significant raw measures, for PD, the highest OR was for time spent inactive, such that one SD increase in the P95 of time spent being inactive is related to a 78% increase in risk for a PA. For DEP, the highest OR was for time spent doing high-intensity activity, where a one SD increase is related to a 67% increase in risk for a PA. For ND, the average step count had the highest OR, where a one SD increase raises the PA risk by 38%. Within the significant Oura scores, for PD, the highest OR was for variation in the Move Every Hour Score, such that a one SD increase was associated with a 55% increase in risk for a PA. For individuals with DEP, the highest OR was observed for the variation in the Stay Active Score, where a one SD increase was associated with a 62% higher risk of a PA. In the ND group, the highest OR was for the 95th percentile of the Sleep Latency Score, where a one SD increase corresponded to a 66% increase in PA risk. Supplementary Figure S2 provides additional information about each feature's significant association with PA across different data aggregation windows across mental health diagnostic groups.

### 3.3 Strength of association depends on feature and amount of data prior to panic attack

**Figure 4:**
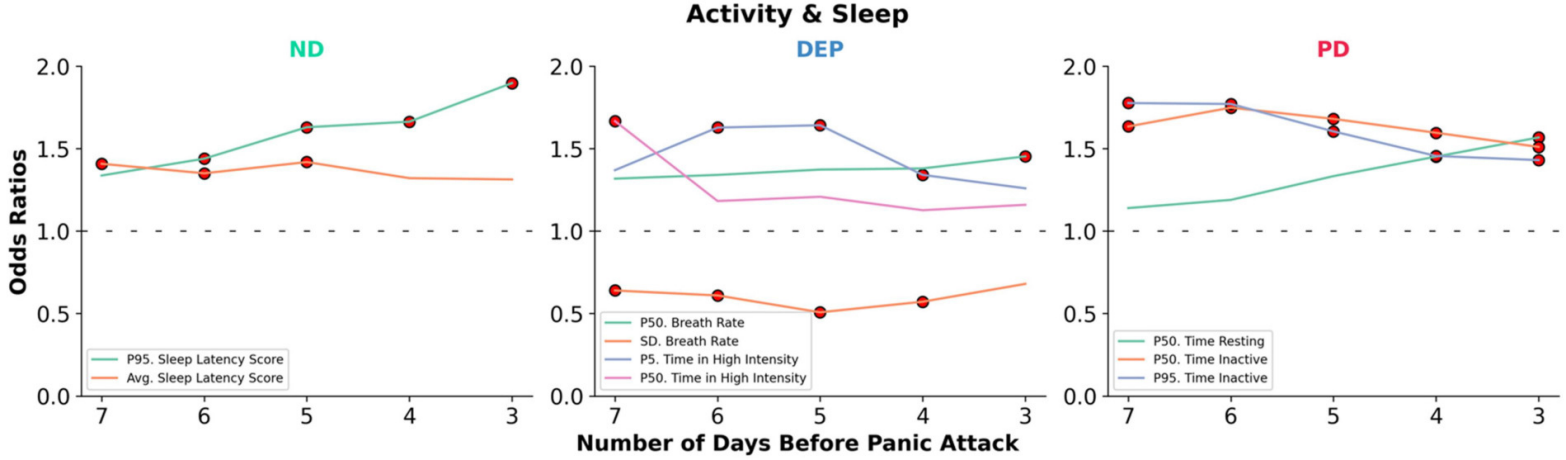
Changes in feature associations with panic attack risk by proximity and mental health diagnosis. Odds ratios (OR) of features with the largest ORs across 3–7 day data aggregation windows are shown for each mental health diagnostic group (PD, DEP, ND). The point of highest magnitude in each trend highlights the window where the association with next-day panic attack risk is the strongest for a given mental health condition. The results indicate a 3-day window as the most relevant for ND, 5–7 days for DEP, and 6–7 days for PD. The red dots (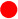) indicate features significantly associated with panic attack risk (*p* < 0.05) (Avg, mean/average; SD, standard deviation; P5, 5th percentile; P50, 50th percentile; P95, 95th percentile).

We further analyzed the trends in ORs of the features across the 3–7 day windows immediately before the PA for each group to elucidate how ORs change based on the amount of data considered before the attack. For this, we iteratively extracted the features that had the highest ORs across each day of aggregation for each mental health group and then plotted the trends of those features that were selected. As shown in Figure 4, the selected top features had the largest OR using a data window of 3 days before a PA for those with ND (Sleep Latency Score), 5–7 days for those with DEP (time spent in high-intensity activity, breath rate), and 6–7 days for those with PD (time inactive).

### 3.4 Multivariable analysis confirms unique associations by diagnostic group

Lasso regression was used to select the most relevant sleep and activity raw measures significant in the univariate models to include in the final multivariable GLMMs for identifying associations with PAs within each diagnostic group (Table 3). For PD, P95 of the time spent staying inactive was positively associated with PAs, where a 1 SD increase in the 95th percentile of the time in inactivity was related to a 76% higher likelihood of experiencing a PA. For DEP, three measures from sleep [P5 Wake-Up Offset, SD Breath Rate, Average Time in rapid eye movement (REM) Sleep] were protective against PAs, and the feature with the highest OR showed that a 1 SD increase in the variability in breathing rate was associated with a 54% decrease in odds of a PA. An activity measure (P5 Time in High-Intensity Activity) had the highest OR where a 1 SD increase in P5 of the time spent in high-intensity workouts was related to a 56% increase in the risk of experiencing a PA. This suggests that if an individual spends 1 SD more time performing high-intensity activity compared with their average, the odds of experiencing a PA increase by 56%. For ND, the significant measure from sleep had a protective effect against PAs, where average HRV defined by the root mean square of successive differences had the highest OR, in which a 1 SD increase was related to a 27% decrease in the likelihood of experiencing a PA. These odds ratios are comparable to those reported in prior research on mental health outcomes using wearable and smartphone data, supporting the plausibility and relevance of these findings (65, 66).

**Table 3:** Multivariable models identifying associations between Oura measures and PA onset by group.

Groups	Sleep/activity	Features	*N* days	Estimate	OR	CI lower	CI upper	Adjusted *p*
PD	Activity	P95. Time inactive	6	0.57	1.76	1.26	2.50	0.013
DEP	Sleep	P5. Wake-up offset	7	−0.42	0.66	0.44	0.97	0.035
	Sleep	SD. Breath rate	5	−0.77	0.46	0.28	0.76	0.010
	Sleep	Average Time in REM sleep	7	−0.6	0.55	0.35	0.86	0.013
	Activity	P5. Time in high intensity	5	0.44	1.56	1.12	2.18	0.013
ND	Sleep	Average HRV (RMSSD)	4	−0.32	0.73	0.55	0.96	0.026

Average, mean/average; SD, standard deviation; P5, 5th percentile; P50, 50th percentile; P95, 95th percentile.

### 3.5 Self-reported stressors before panic attacks differ by mental health diagnosis

**Figure 5:**
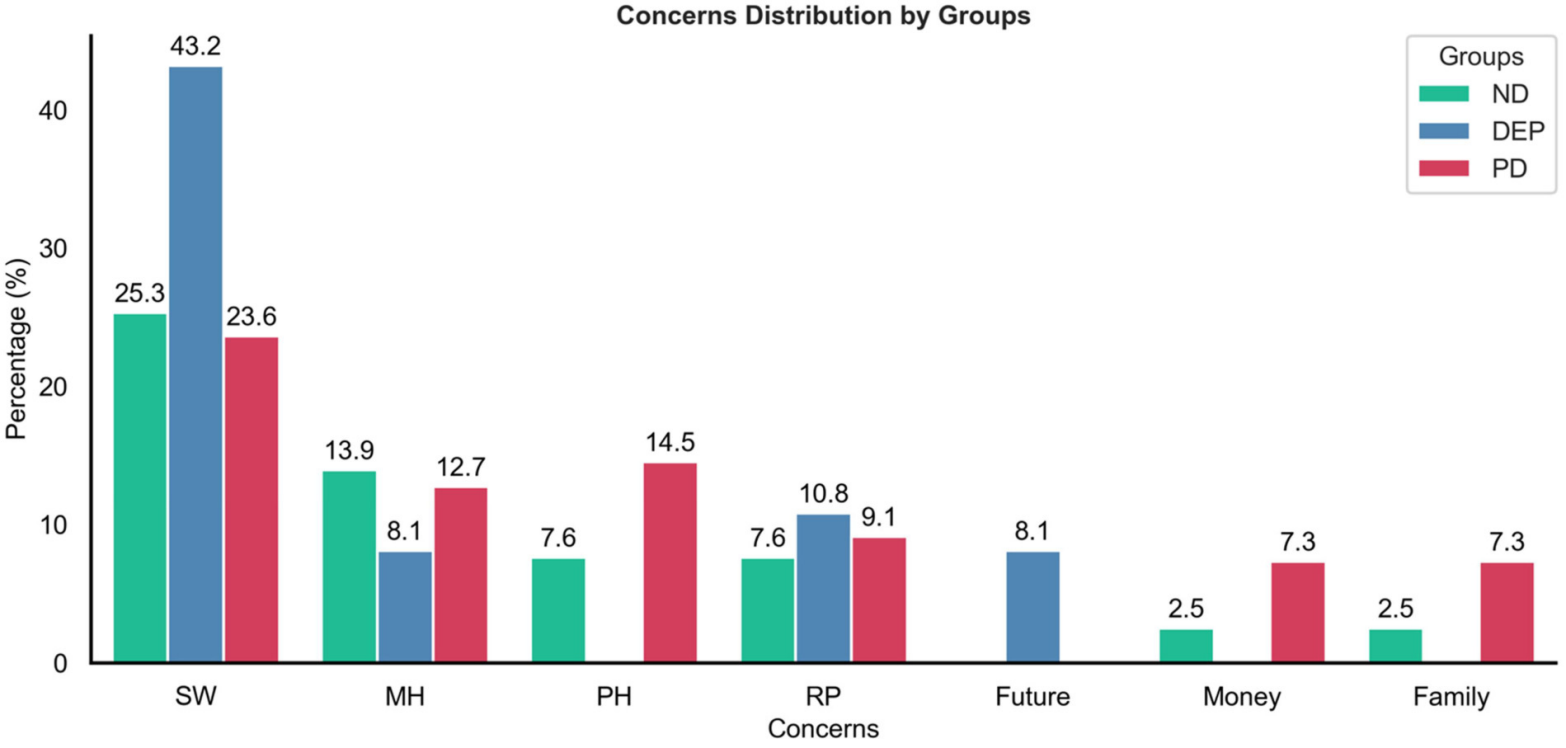
Stressors reported before panic attacks. A content analysis of the top concerns reported by each of the mental health diagnostic groups on days when a panic attack was reported reveals that most stressors were related to SW across all groups. Differences emerged between groups in the relative prevalence of stressors related to schoolwork, physical health (PH), and the future. SW, schoolwork; MH, mental health; PH, physical health; RP, romantic partner.

In addition to objective measures from passive sensing, we consider participant-reported stressors. A content analysis revealed that the PD group paralleled ND in the topic and order of their top six biggest concerns reported on PA days, except that PD reported 25% more concerns overall than NDs. DEP was differentiated from ND and PD by having no concerns regarding physical health, some concerns about the future, and relatively more concerns about schoolwork (SW) (Figure 5). Average number of daily stressors associated with PA risk for those with PD, where a 1 SD increase in the number of stressors increased the likelihood of a PA by 67%, which had the most impact when a 7-day aggregate window prior to a panic attack was used.

## 4 Discussion

PAs occur across mental health diagnoses, yet research on the precursors of PAs has largely been limited to PAs experienced by those with panic disorder. As groups with heightened risk for PAs (e.g., those with childhood adversity, depression, and panic disorder) have distinct patterns of daily behavior, physiology, and stress reactivity, it follows that they may also have distinct profiles preceding PA onset. In this work, we explored PA onsets across multiple risk groups for the first time. We examined wearable sensor–derived measures of behavior and physiology leading up to PAs for individuals with ACEs compared with those without, and for individuals with panic disorder and depression compared with those with no mental health conditions.

First, in our sample of only individuals who had experienced at least one PA on study, most (84%) reported an ACE. Individuals with ACEs, compared with those without, reported significantly more PAs, highlighting the lasting impact of childhood trauma on mental distress during the transition to adulthood (5, 38, 67–72). There were very few common features linked to PA risk between these groups (Figure 2). Those with ACEs had many features related to impending PAs, each with a small effect, potentially suggesting hypervigilance and sensitivity to physiological and behavioral triggers (68, 73), compared with those without, and also, a likely unexplained heterogeneity within the group.

To address this heterogeneity, we subsequently compared groups by current mental health diagnosis. Individuals with a depressive disorder only, those with panic disorder, and those without any mental health condition were examined. Interestingly, the rates of panic attacks (5.8% vs. 5.6% vs. 4.8%, respectively) were similar across diagnostic status, supporting continued PA research outside of only panic disorder. There were no features related to PAs overlapping across diagnostic groups (Table 1). For those with PD, time spent physically inactive across the past 6 days was associated with PA onset. We speculate that large periods of inactivity may be indicative of overwhelming anxiety and activity avoidance and/or potential overconsumption of online content while inactive, termed “brain rot” (74) or “doomscrolling” (75). For those with depression, time in high-intensity physical activity appeared to have an impact on PA onset such that individuals not allowing themselves a rest day from high-intensity physical activity may be pushing themselves too hard (76–78). This message of moderating activity may be especially salient to relay in PA treatments for those with depression, as current interventions often focus on the robust benefits of exercise on mental health (79–83). Variation in respiratory rate was also associated with PAs, consistent with prior work (84–86) linking respiratory patterns to PA risk and identifying respiration as a target for interventions (49, 87, 88). Notably, this finding extends previous evidence from populations with panic disorder (PD) to individuals with depression. Finally, for those with depression, sleep features were associated with PAs, including earlier wake-up times and less time in REM sleep, which, in the context of a college student may be indicative of a more stressful academic course schedule (89). There is some research connecting sleep stages to PAs (48), which may be exacerbated in those with depression (90), a condition strongly linked to sleep disturbances (91). For those with no diagnoses, lower HRV, a feature generally linked to stress (92) and poor mental health (93, 94), was associated with PAs, consistent with past literature on autonomic nervous system activity in those with PD (28) and with an emphasis on biofeedback interventions for anxiety (95) and panic (16, 20).

Aside from passively collected behavioral and physiological data, self-reported stressors were associated with a risk of PAs for those with PD only (32). Individuals with PD reported up to 25% more “biggest concerns” than other diagnostic groups, which was consistent with past literature (81). However, as self-reported stressors did not show statistically significant associations with PAs for other groups, passive data from wearables may be important to collect in future studies to inform PA risk when investigating populations with diverse mental health histories.

Features significantly associated with PAs differed in the amount of data aggregated from 3 to 7 days before a panic attack. The results suggest that features may be most impactful when more days of data are used for those with PD and depression and fewer days for those with no diagnoses (Figure 4). However, it is unknown whether the amount of data required is indeed related to diagnostic group type or severity, or is simply feature dependent, as there was no overlap between significant features across groups. Notably, the multiple days of aggregate data required to optimally assess PA risk may help us understand why PAs are described as “unexpected” and “unpredictable” by those suffering the most (96–98). For example, it appears that there is no one triggering event that leads to a PA for those with PD; rather, it is the cumulative inactivity across the past week that increases the likelihood of a PA, which may be challenging to identify retrospectively.

Although challenging to replicate because of proprietary algorithms, scores developed by Oura seem to exhibit value in estimating PAs across risk groups. Specifically, a unit increase in sleep latency scores for those without diagnoses, variation in a score related to avoiding long periods of inactivity for those with PD, and variation in a score related to avoiding inactivity for those with depression each appear to increase the likelihood of a PA by more than 50% (OR > 1.5, Figure 3). These features could be immediately and directly useful for Oura users suffering from PAs and potentially inform timely interventions to mitigate risk.

While these models could help future researchers inform hypotheses to study PA risk in more diverse samples, and potentially inform individuals in their management of their own PAs, there are a number of limitations in this study. In this analysis, we explored three diagnostic groups; however, we excluded most individuals with alternate mental health diagnoses and comorbidities. Thus, we must caution against any generalizability of these findings, which should be replicated in samples with better representation of the realities of having mental health diagnoses (i.e., those with ADHD, anxiety and other comorbidities). Similarly, the data considered in this work were obtained from a sample of college students with relatively homogeneous demographics, despite the fact that they had high mental health impairment (38% of the LEMURS cohort endorsed experiencing at least one PA during the study), which also likely impacts the frequency and type of childhood traumatic events. While these data provide value for understanding the physiological and behavioral markers of PAs, future studies would benefit from considering participants with a wider range of ages, genders, races, and ethnicities. It is often a challenge in longitudinal studies to understand the context of passively collected data, as there can be general confounding factors (e.g., time of year, day of week, and weather) and sample-specific confounding factors (e.g., exam schedule and semester break schedules) that impact behaviors. While a detailed examination of potential confounding factors was beyond the scope of this work, future work may benefit from adding these factors as covariates. Because we used ecological daily assessments for reporting panic attacks, we also acknowledge that there is a possibility for recall bias; hence, future studies may aim to implement ecological momentary assessments or just-in-time approaches to obtain panic event data. The participants in this study also had access to their wearables data in real time, which could impact subsequent emotion and behavior. In terms of analyses, although the differences in sample size across the PD, DEP, and ND groups were modest, and GLMMs inherently account for such variability through their confidence intervals, we nevertheless restricted all interpretations to within-group associations rather than making any comparisons across groups. Finally, future work could explore integrating multiple features using machine learning approaches to enable adequate performance for forecasting when PAs are likely to occur.

This exploratory work demonstrates that changes in objective physiological and behavioral data measured by wearable sensors are associated with the onset of panic attacks and suggests that future studies continue to investigate these associations by diagnostic status. With replication of these findings, it may be possible to develop accurate prediction models for PAs to inform the timing of future preventive interventions.

## Data Availability

The original contributions presented in the study are included in the article/Supplementary Material, further inquiries can be directed to the corresponding author.
