# Supplementary material for: Exploration of wearable sensor measures associated with panic attacks differs across mental health conditions
Source: Front Digit Health. 2026 Apr 20;8:1764371. doi: 10.3389/fdgth.2026.1764371 (PMC13136185; doi:10.3389/fdgth.2026.1764371)
Supplement: Supplementary file 1 [file Datasheet1.docx]

Supplementary Material

**Table S1**. Adverse Childhood Experiences (ACES) category-wise criteria description. Participants were classified into the ACE group if they reported ACE criteria exceeding or meeting the defined threshold value.

| **Category** | **Criteria** | **Threshold** |
| --- | --- | --- |
| ACES 1 | Did you live with anyone who was depressed, mentally ill, or suicidal? | 1 |
| ACES 2 | Did you live with anyone who was a problem drinker or alcoholic? | 1 |
| ACES 3 | Did you live with anyone who used illegal street drugs or who abused prescription medications? | 1 |
| ACES 4 | Did you live with anyone who served time or was sentenced to serve time in a prison, jail, or other correctional facility? | 1 |
| ACES 6 | How often did your parents or adults in your home ever slap, hit, kick, punch or beat each other up? | 2 |
| ACES 7 | Not including spanking, (before age 18), how often did a parent or adult in your home ever hit, beat, kick, or physically hurt you in any way? | 2 |
| ACES 8 | How often did a parent or adult in your home ever swear at you, insult you, or put you down? | 2 |
| ACES 9 | How often did anyone at least 5 years older than you or an adult, ever touch you sexually? | 1 |
| ACES 10 | How often did anyone at least 5 years older than you or an adult, try to make you touch them sexually? | 1 |
| ACES 11 | How often did anyone at least 5 years older than you or an adult, force you to have sex? | 1 |

**Table S2.** Distribution of Participants by Racial/Ethnic Group

| **Race/Ethnicity** | **n(%)** |
| --- | --- |
| Hispanic | 17(9%) |
| Black/African Caribbean | 3(2%) |
| Asian heritage | 7(4%) |
| Middle Eastern | 7(4%) |
| Native American | 1(.5%) |
| Other race | 11(6%) |
| White | 136(74%) |
| **Total** | 182(100%) |

Note. For individuals who identified with multiple racial/ethnic categories, classification was based on their membership in the least represented racial/ethnic group to maximize representation of demographic diversity.

**Table S3.** Sensitivity analysis: evaluating the changes in odds ratios at different levels of interpolation for the features that remained significant after applying multivariable models across each mental health diagnostic group. Associations remain signficiant and the directions are consistent across levels of interpolation.

| Group | Feature | Interpolation level | # Number of Observations | OR | p |
| --- | --- | --- | --- | --- | --- |
| PD | P95. Time Inactive | 3 days | 718 | 1.76 | 0.001 |
|  |  | 2 days | 669 | 1.82 | <0.001 |
|  |  | 1 day | 635 | 1.61 | 0.01 |
| DEP | P5. Time in High Intensity | 3 days | 601 | 1.57 | 0.01 |
|  |  | 2 days | 566 | 1.7 | <0.001 |
|  |  | 1 day | 536 | 1.5 | 0.04 |
| ND | Avg. HRV (RMSSD) | 3 days | 1223 | 0.73 | 0.03 |
|  |  | 2 days | 1154 | 0.74 | 0.04 |
|  |  | 1 day | 1048 | 0.68 | 0.02 |


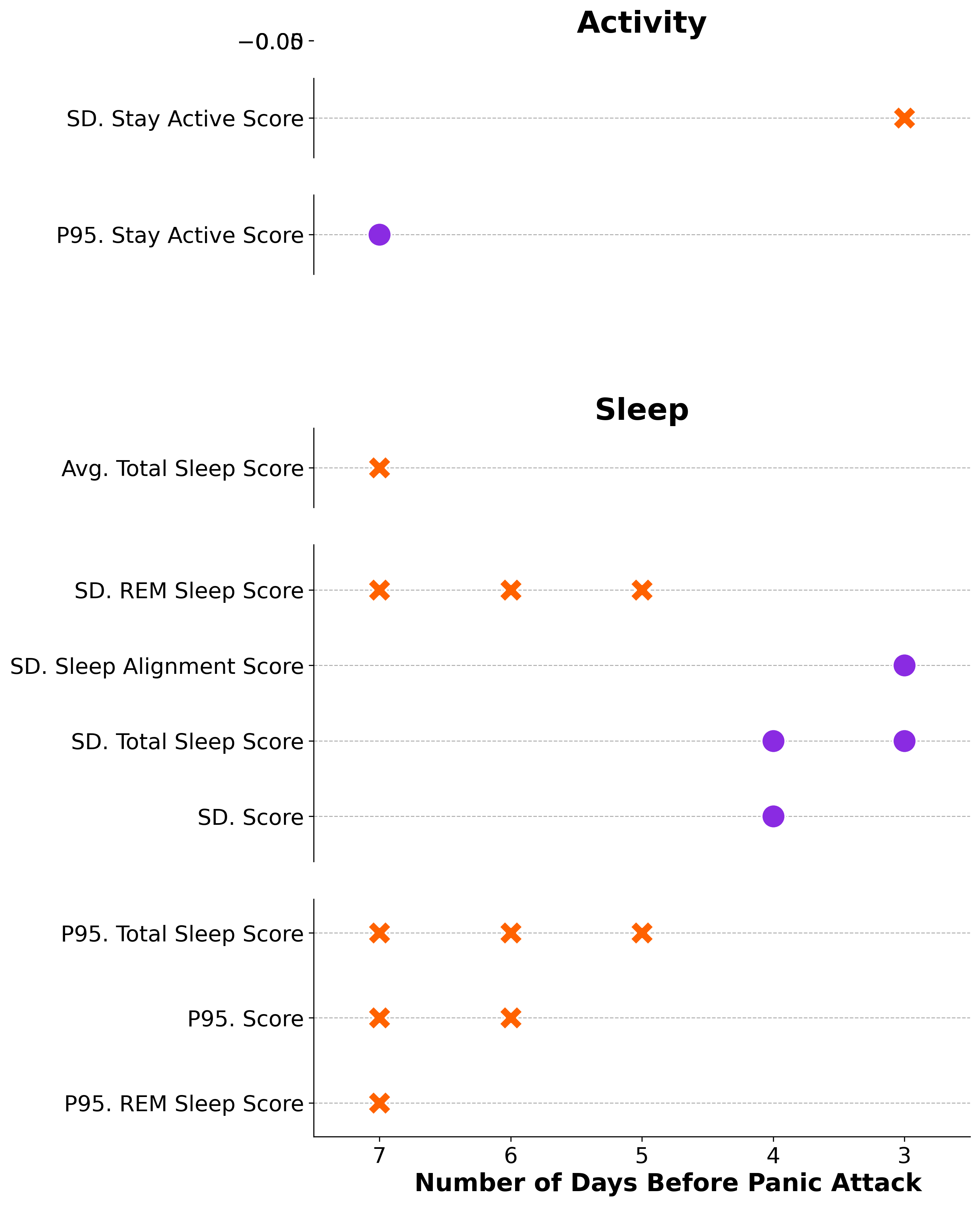

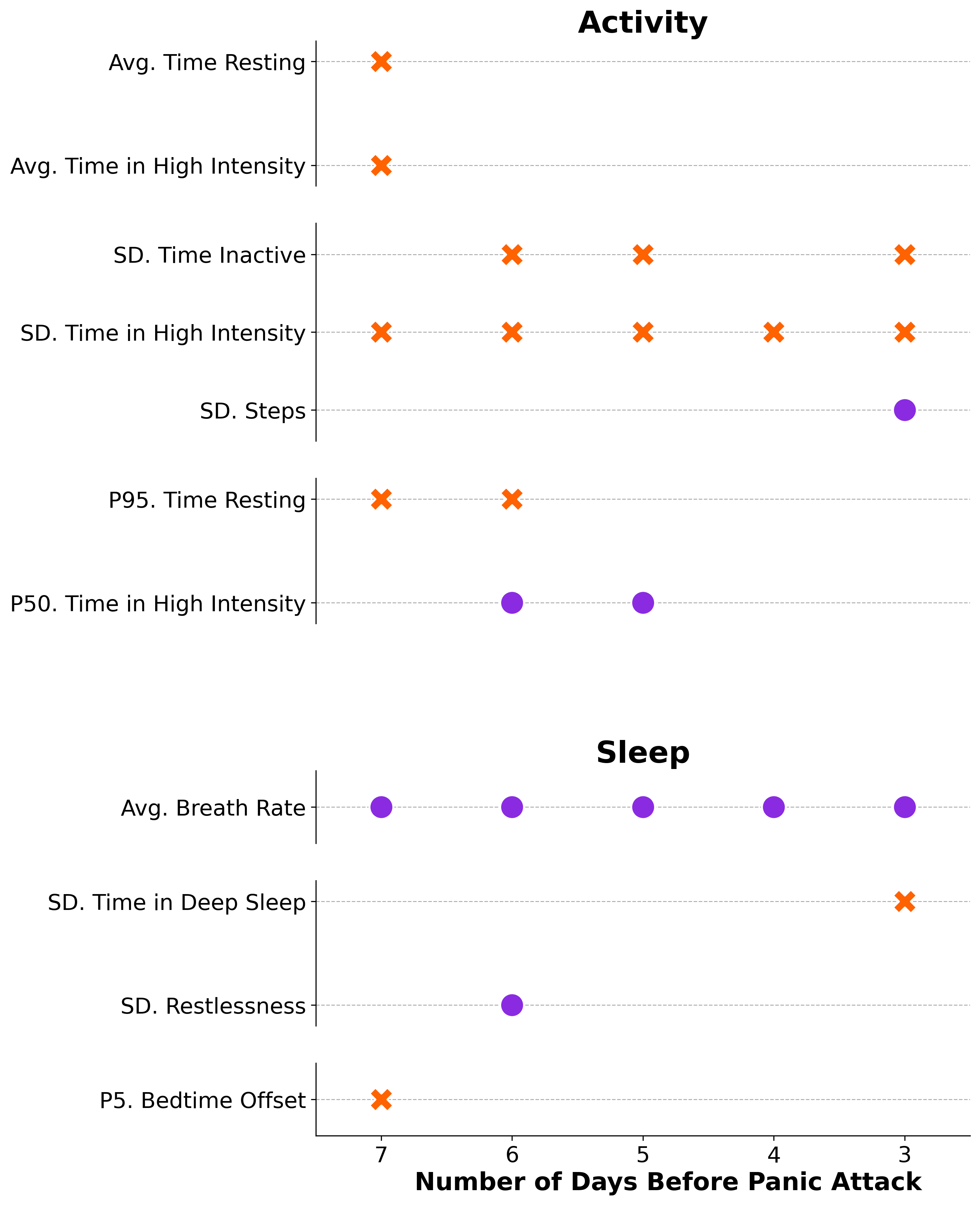


**Fig S1. Associations Between Features and Panic Attack Risk Across Data Windows and Mental Health Risk.** Significant (p < 0.05) features associated with next-day panic attacks for each mental health risk group (ACE – orange vs. None – purple) are reported across data aggregation windows of 3–7 days prior to a panic attack. Features are shown separately for raw Oura measures (left) and Oura-derived scores (right). Aggregation windows shorter than 3 days were excluded due to insufficient data for reliable statistical estimation.


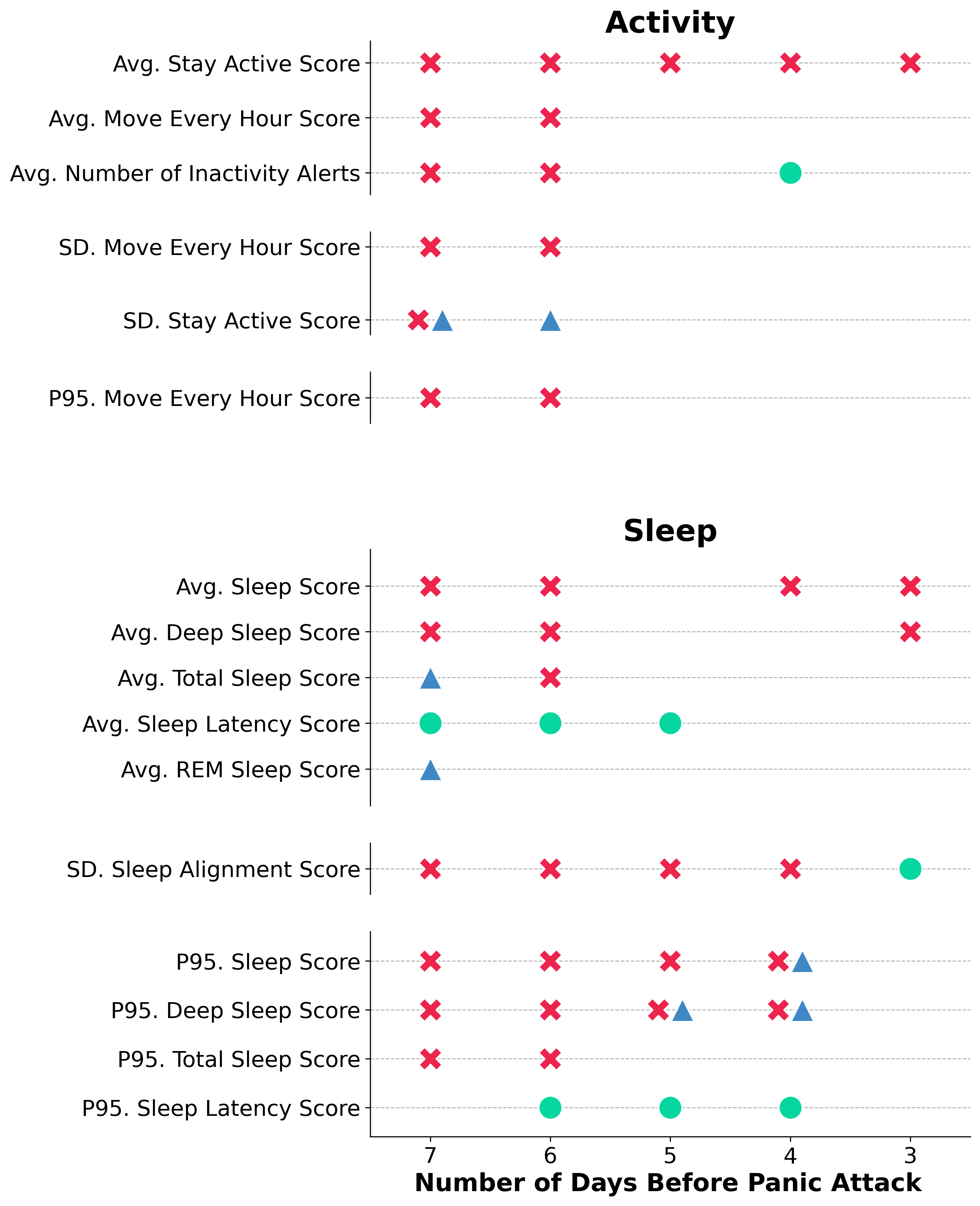

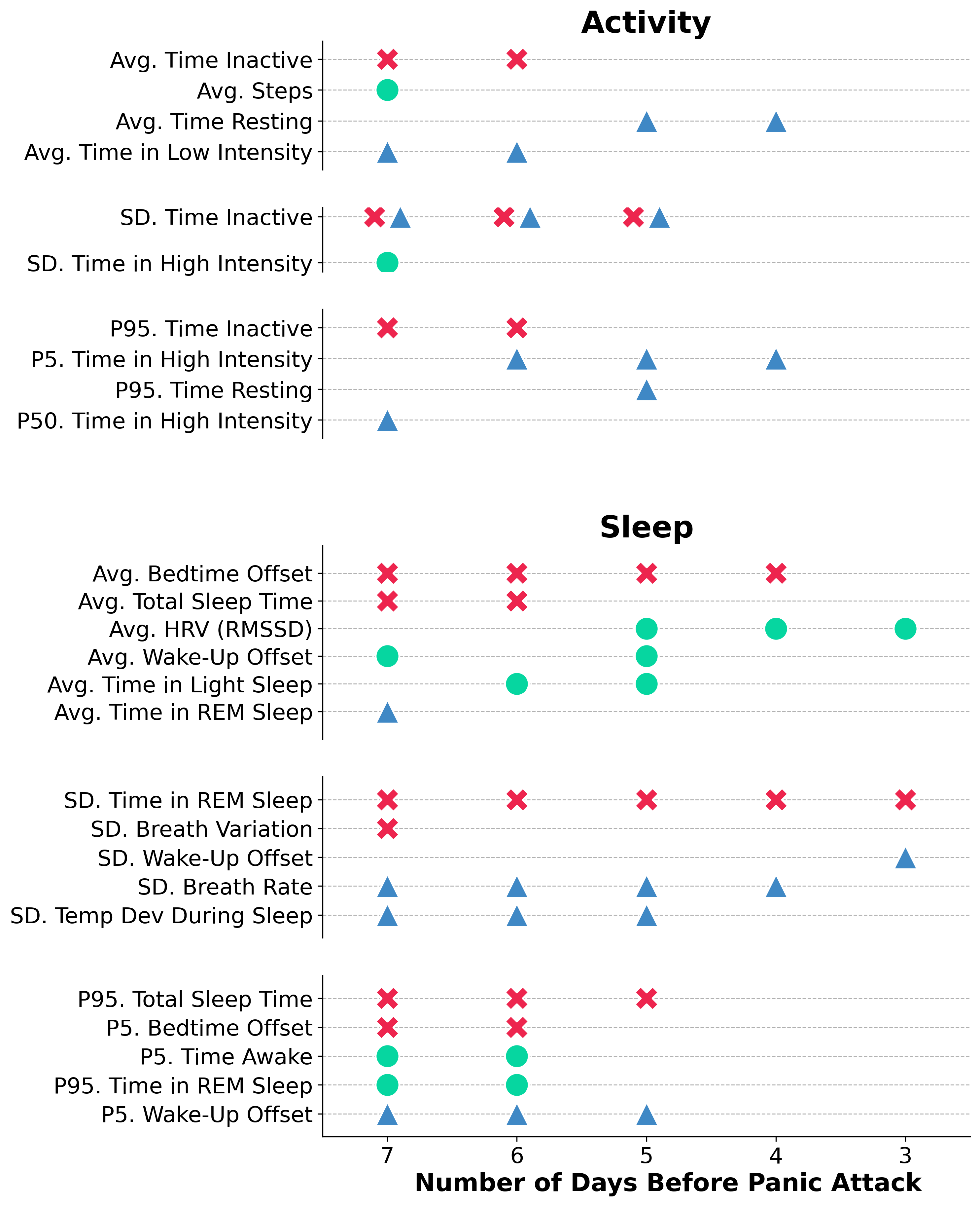


**Fig S2. Associations Between Wearable Features and Panic Attack Risk Across Data Windows and Mental Health Diagnosis.** Significant (p < 0.05) features associated with next-day panic attacks for each mental health disorder group (ND – green, DEP – blue, PD – red) are reported across data aggregation windows of 3–7 days prior to a panic attack. Features are displayed separately for raw Oura measures (left) and Oura-derived scores (right). Aggregation windows shorter than 3 days were excluded due to insufficient data for reliable statistical estimation.

***
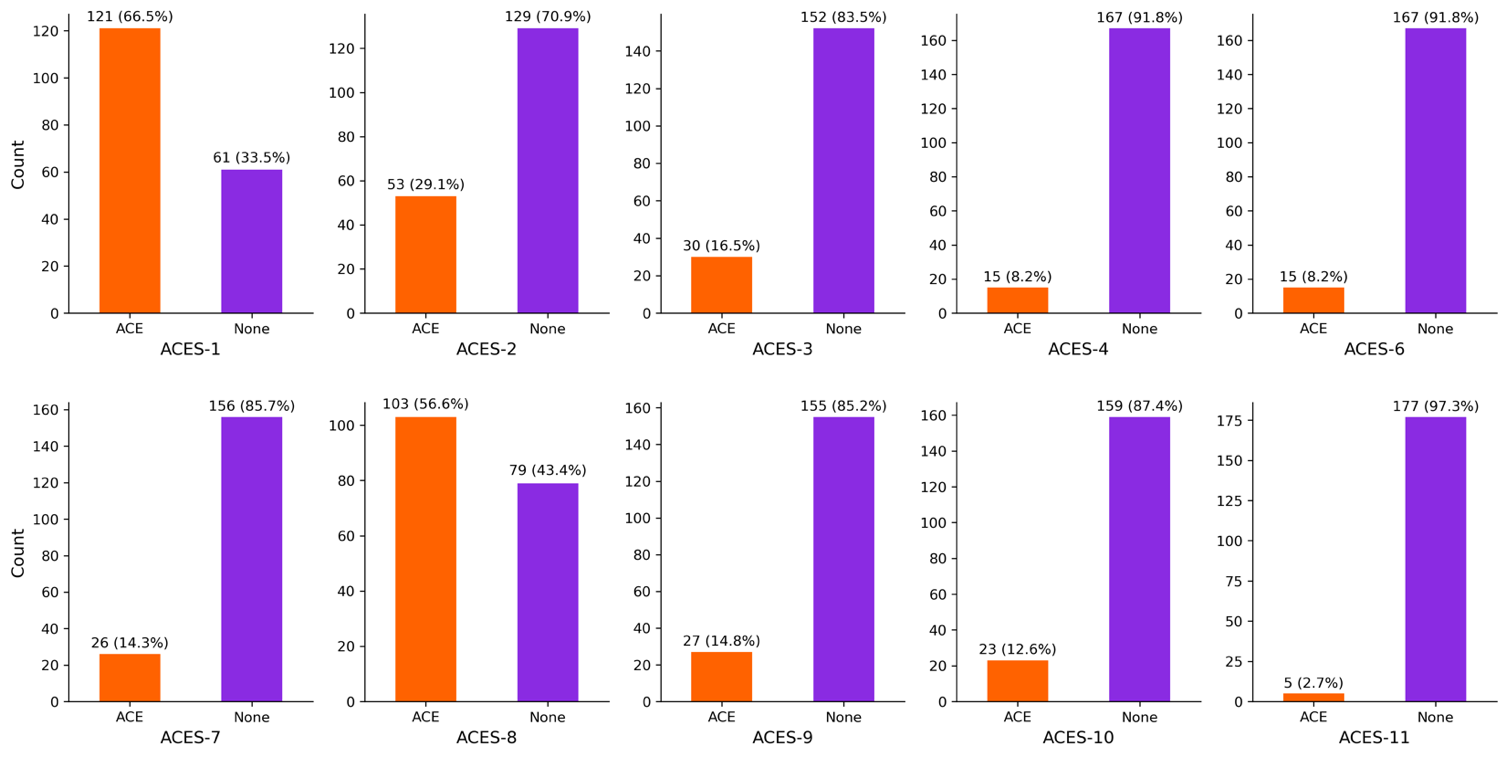
***

**Fig S3. Distribution of ACEs.** Distribution of sample across the ACEs categories. The higher prevalence of participants belonging to the ACE group is explained by the preselection of candidates who reported at least one panic attack.

*
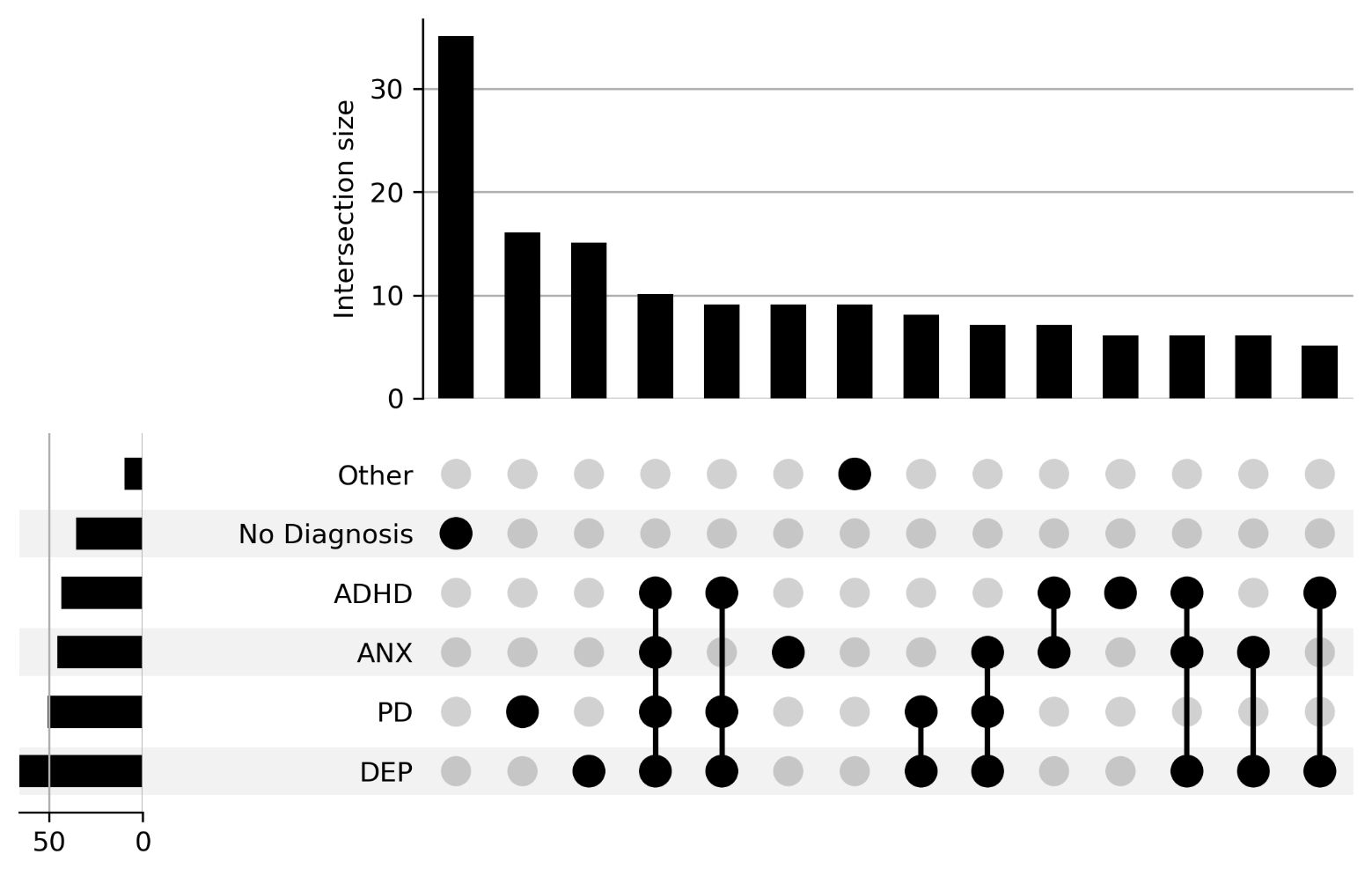
*

**Fig S4.** **Participant Comorbidities.** Distribution of mental health diagnoses and comorbidities in the sample. DEP: Depression, PD: Panic Disorder (without and with comorbid ANX and/or ADHD), ANX: General Anxiety Disorder, ADHD: Attention Deficit Hyperactivity Disorder, No Diagnosis are people with no mental health condition, and Other: other mental health conditions (e.g. alcoholism, anorexia, bipolar disorder).
